# The potential effect of *Moringa oleifera* ethanolic leaf extract against oxidative stress, immune response disruption induced by abamectin exposure in *Oreochromis niloticus*

**DOI:** 10.1007/s11356-023-26517-0

**Published:** 2023-03-29

**Authors:** Rasha M. Reda, Rania M. A. Helmy, Ali Osman, Farag A. Gh. Ahmed, Gamila A. M. Kotb, Amir H. Abd El-Fattah

**Affiliations:** 1 Department of Aquatic Animal Medicine, Faculty of Veterinary Medicine, Zagazig University, P.O. Box 44511, Zagazig, Egypt; 2 Pesticides Residue and Environmental Pollution Department, Central Agricultural Pesticides Laboratory, Agricultural Research Center, Dokki, Giza, 12618 Egypt; 3 Biochemistry Department, Faculty of Agriculture, Zagazig University, P.O. Box 44511, Zagazig, Egypt; 4 Plant Protection Department, Faculty of Agriculture, Zagazig University, P.O. Box 44511, Zagazig, Egypt; 5 Mammalian and Aquatic Toxicology Department, Central Agricultural Pesticides Laboratory, Agricultural Research Center, Dokki, P.O. Box 12618, Giza, Egypt; 6 Department of Animal Wealth Development, Faculty of Veterinary Medicine, Zagazig University, P.O. Box 44511, Zagazig, Egypt

**Keywords:** *Moringa oleifera*, Abamectin, Tilapia, Residues, Anti-inflammatory gene, Antioxidant, Immunity

## Abstract

Abamectin (ABM), a naturally fermented product of *Streptomyces avermitilis*, is applied to pest control in livestock and agriculture fields. The aim of the current study is to evaluate the protective effects of *Moringa oleifera* leaf ethanolic extract (MOE) on biochemical changes including oxidative stress indices, immune response marker, lipid profiles as well as mRNA expression of immune related genes, and abamectin (ABM, 5% EC) residue levels in Nile tilapia (*Oreochromis niloticus*) exposed to a sub-lethal concentration (0.5 µg/l) for 28 days. Disturbance in liver and kidney biomarkers was markedly increased in ABM-exposed fish compared to the control group. Malondialdehyde levels in the liver and brain tissues, as well as the activities of glutathione-s-transferase, superoxide dismutase, and glutathione peroxides, all increased significantly in ABM group. Additionally, ABM exposure increased the levels of interleukin 10 beta and growth factor gene expression. On the other hand, fish exposed to ABM had significantly lower serum alkaline phosphatase, creatinine, high-density lipoprotein, glutathione peroxides in brain, glutathione in liver and brain tissues, lysozyme activity, nitric oxide, immunoglobulin M, tumor necrosis factor, and interleukin 1 beta as compared to the control group. The recorded detrimental effects of ABM on tilapia have been overcome by the addition of MOE to the diet (1%) and ameliorating hepato-renal damage and enhancing antioxidant activity, innate immune responses, and upregulating the anti-inflammatory gene expression. Therefore, it could be concluded that MOE dietary supplementation at 1% could be used to counteract the oxidative stress, immune response disruption induced by abamectin exposure in *Oreochromis niloticus,* and reduce its accumulation in fish tissues.

## Introduction

Several pollutants are present in our environment and exert various kinds of stress on the ecosystem and can be proven to be deleterious to organisms (Abu Zeid et al. 2021; El-Bouhy et al. 2021; Galal et al. 2018; Khalil et al. 2017; van der Oost et al. 2003). Abamectin (ABM) belongs to the group of drugs known as avermectins, which are produced naturally as a byproduct of the fermentation of *Streptomyces avermitilis*, an actinomycete identified from soil (Batiha et al. 2020). The ABM is popular among farmers and veterinarians due to its broad spectrum of activity, ease of use, and wide margin of safety for the targeted animals. ABM is currently an active component of various insecticides and nematocidal treatments used in agriculture, as well as one of the most often used medicines in veterinary medicine for several years in the prevention of parasitic infections (Hedayati et al. 2014; Santos et al. 2023; Vajargah et al. 2021). Because of its slow degradation and lipophilic nature, ABM residues can access aquatic habitats through runoff or drift and accumulate in fish and aquatic bodies (Kolar et al. 2008). ABM has been categorized as extremely harmful to some aquatic lives with long-lasting consequences because it can cross the blood and brain barrier in some aquatic species and accumulate in fish (Novelli et al. 2016, 2012; Wang et al. 2011). ABM toxicological properties are due to its two active components, avermectin B1a (80%) and avermectin B1b (20%), which impact inhibitory synapses via a mechanism involving glutamate-sensitive chloride channels, impairing neuronal coordination. (González Canga et al. 2009; Santos et al. 2023; Yoon et al. 2004). Furthermore, the toxic effects of ABM may include a disturbance in the functioning of the liver and kidneys, as well as a change in hematological parameters and vital physiological processes (Kushwaha et al. 2020; Salman et al. 2022).

Nutritional supplements are now included in aquatic feed to aid the body’s systems in processing and absorbing the nutrients obtained from the food consumed and to improve fish health (Reda et al. 2016; Reda and Selim 2015; Selim and Reda 2015). Despite the existence of many research studies that proved the efficiency of medicinal plants in protecting fish from the toxic effects of many pollutants on the aquatic environment and helped to improve the state of health and immunity, the studies that discussed the effect of medicinal plant protection against pesticide toxicity are limited (Abu Zeid et al. 2021; El-Bouhy et al. 2021; Farag et al. 2022, 2021; Khalil et al. 2017). Due to its low cost, local availability, and high protein content, recent studies have focused on the use of Moringa leaf in aquafeeds (Abdel-Latif et al. 2022; Brar et al. 2022). The genus Moringa belongs to the Moringaceae family of plants. In Southwest Asia, Southwest Africa, Northeast Africa, and Madagascar, there are 13 species of the Moringa genus. Only four of the thirteen species are the subject of current research (*Moringa oleifera*, *Moringa stenopetala*, *Moringa concanensis*, and *Moringa peregrina*) (Abd Rani et al. 2018). *Moringa oleifera* Lam is the most prominent species of the Moringa genus; it contains a variety of beneficial phytochemical substances such as phenolic and flavonoid compounds (Sankhalkar and Vernekar 2016), alkaloids, tannins, triterpenoids, sterols, isothiocyanate (Leone et al. 2015), and moringyne (Dalei et al. 2016; Ganatra et al. 2012; Oyedara et al. 2021) with potent anthelmintic (Hegazi et al. 2018), antiviral (Biswas et al. 2020; Xiong et al. 2022), and antibacterial effects (Anthony et al. 2017; El-Kholy et al. 2018; Fouad et al. 2019).

Even though the effects of Moringa leaf extract (MOE) on fish health and growth performance have been studied (Abdel-Latif et al. 2022; Abidin et al. 2022; El-Kassas et al. 2022), little is known about how MOE can mitigate the toxicological effects of ABM on *Oreochromis niloticus* (*O. niloticus*)*.* Therefore, the evaluation of hepatic-renal toxicity, lipid profiles, immunological parameters and immune-related gene, and antioxidant capacity, and detailed descriptions of potential bioaccumulation in brain, gills, liver, kidney, and muscles of *O. niloticus* during ABM exposure alone or in combination with MOE are the main objectives of this study.

## Materials and methods

### Moringa leaf ethanolic extract (MOE) preparation and characterization

*Moringa oleifera* leaves were obtained from National Research Centre (NRC), Dokki, Giza, Egypt, cleaned, and processed, and the meals were crushed to pass through a 1-mm^2^ sieve. The powder was then defatted with N-hexane for 8 h. The ethanolic *Moringa oleifera* leaf extract (MOE) was prepared according to Abdel-Shafi et al. (2019) and Omar et al. (2020). Defatted *Moringa oleifera* leaf powder (100 g) was extracted with 70% v/v aqueous ethanol for 2 h. One hundred grams of *Moringa oleifera* leaf powder yielded a 20 g ethanolic extract. The total phenolic contents (TPCs) of MOE (10 mg/10 ml) was determined using the Folin–Ciocalteu method published by Singleton et al. (1999). Gallic acid was diluted in distilled water at several concentrations (25–250 µg/ml) to obtain standard curve. The calibration equation for gallic acid was *y* = 0.0093*x* − 0.00224 (*R*^2^ = 0.9948), where *y* and *x* are the gallic acid absorbance and concentration in µg/ml, respectively. Total flavonoids (TFs) of the MOE (10 mg/10 ml) was estimated according to the protocol of Ordoñez et al. (2006) as described in Abdel-Shafi et al. (2019). Quercetin was diluted in ethanol at several concentrations (10–100 µg/ml) to obtain standard curve. Total flavonoid contents were stated as quercetin equivalent (QE), which was calculated based on the calibration curve: *y* = 0.0115*x* − 0.0119 (*R*^2^ = 0.994), where *y* is absorbance and *x* is concentration of quercetin in µg/ml. The identification and quantification of phenolic compounds and flavonoids in MOE are typically conducted using HPLC (Agilent 1100 series) analysis according to the method described by Osman and Elsobki (2019). The Eclipse Plus C18 column (4.6250 mm, i.d. 5 m) was used to separate the samples. At a flow rate of 1 ml/min, the mobile phase comprised of water (A) and 0.02% tri-floro-acetic acid in acetonitrile (B). An elution gradient program was applied as follows: 0 min (80% A), 0–5 min (80% A), 5–8 min (40% A), 8–12 min (50% A), 12–14 min (80% A), 14–16 min (80% A), 14–16 min (80% A), 14–16 min (80% A), 14–16 min (80% A), 14–16 min (80% A), and 14–16 min (80% A) (80% A). At 280 nm, the multiwavelength detector was monitored. The injection volume was 10 l. The temperature of the column was kept at 35 °C. The active components of the extract were identified by comparing the peak area of the samples to those of the standards.

### Pesticide

**Fig. 1:**
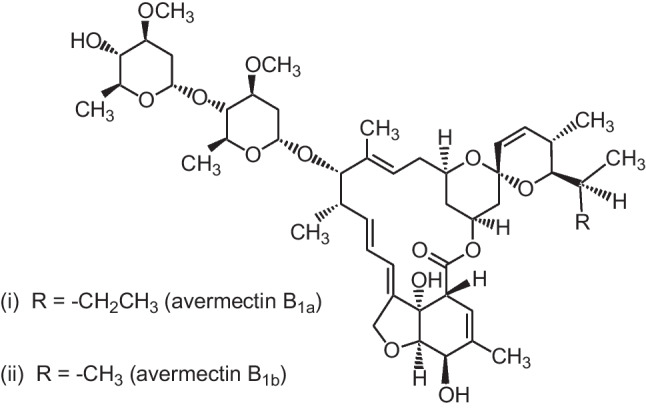
Chemical structure of abamectin

Abamectin (ABM 5% EC, Profery) was provided by the Central Agricultural Pesticide Laboratory (CAPL), Agricultural Research Center (ARC), Dokki, Giza, Egypt. ABM chemical name is 5-O-demethylavermectin A_1a_ (i) mixture with 5-O-demethyl-25-de(1-methylpropyl)-25-(1-methylethyl) avermectin A_1a_ (ii) (Fig. 1). The physical characteristics of the ABM are that it is colorless to pale yellow crystals. The LC_50_–96 h of ABM was previously estimated in our laboratory and found to be 10 µg/l (Farag and Reda 2021). In this study, we will apply 1/20 of LC_50_-96 h of ABM (0.5 µg/l).

### Diet preparation and experimental design

Two experimental diets were prepared in Fish Research Unit, Faculty of Veterinary Medicine, Zagazig University. The first is the control reference diet (R-D), which was prepared to meet the nutrient requirement of *O. niloticus* according to National Research Council (NRC 2011) (Table 1). The second diet (MOE-D) was supplemented with 1% MO leaf ethanolic extract (MOE) (Ahmed et al. 2020). Mechanical mixing of the ingredients was followed by pellet preparation using a pellet machine with a 1.5-mm particle diameter. The pellets were air dried at 27 °C for 24 h before being stored in a refrigerator at 4 °C until use.

**Table 1:** Composition of the experimental diets (%)

Diet ingredients (%)	Diets	
	*R-D	**MOE-D
Fish meal (65.4% CP)	40	40
Soybean meal (44%)	20	20
Yellow corn	13	13
Wheat flour	15	15
Wheat Bran	2	2
Fish oil	7	7
Monocalcium phosphate	2	2
^(1)^ Vitamin mixture	0.45	0.45
^(2)^ Mineral mixture	0.55	0.55
*Moringa oleifera* leaf extract	-	1
Chemical analyses (% DM)		
Crud protein	38.90	38.90
Crude fat	10.50	10.79
Ash	5.84	5.84

^*^R-D = control reference diet (NRC 2011)

^**^ MOE-D = control reference diet supplemented with 1% *Moringa oleifera* leaf extract

^(1^^)^Vitamin mix (IU or mg kg diet): vitamin A, 16,000 IU; vitamin D, 8000 IU; vitamin K, 14.72; thiamin, 17.8; riboflavin, 48; pyridoxine, 29.52; cynocobalamine, 0.24, tocopherols acetate, 160; ascorbic acid (35%), 800; niacinamide, 79.2; calcium-D- pantothenate,73.6; folic acid, 6.4; biotin, 0.64 L-carnitine, 100

^(2)^ Mineral mix (mg kg diet): Cu (CuSO4), 2.0; Zn (ZnSO4), 34.4; Mn (MnSO4), 6.2; Fe (FeSO4), 21.1; I (Ca (IO3)2), 1.63; Se (Na2SeO3), 0.18; Co (CoCl2), 0.24; Mg (MgSO4.H2O), 52.7

A total of one hundred and twenty fish, apparently healthy *O. niloticus* (80 ± 10 g), were obtained from a private fish farm in Kafr El-Sheikh Governorate, Egypt. The experiment was conducted at the Department of Aquatic Animal Medicine, Faculty of Veterinary Medicine, Zagazig University. All fish were acclimated for 2 weeks at least and fed daily at 3% of body weight. Each aquarium was equipped with aeration system, and water physicochemical conditions were maintained according to APHA (2015). Experiment was performed according to OECD (Organization for Economic Co-operation and Development) using fish prolonged toxicity test No. 204 (OECD 1992). After acclimatization, fish were divided into four groups in triplicate (10 fish/replicate). The 1st (CONT) and 2nd (ABM) groups were fed with R-D diet without and with exposure to 0.5 ug/l of abamectin, respectively. The 3rd (MOE) and 4th (ABM + MOE) groups were fed with MOE-supplemented diets without and with exposure to 0.5 ug/l of abamectin, respectively. Test fish were monitored at regular time intervals during the experimental period (28 days), with records kept for clinical symptoms (including swimming behavior and reflexes), mortality, and postmortem findings.

After the testing period, the fish were anesthetized with clove oil at a concentration of 7.4 ml/l (Rezende et al. 2017). The blood samples were taken from the caudal vein (9 samples/group), centrifuged at 3000 rpm for 10 min, separated from serum carefully, collected, and stored at − 80 ℃ for analysis of biochemical and immune parameters. To gather the various organ tissues, the fish were anesthetized and then euthanized by spinal cord severing. Liver and brain samples from nine fish per group were taken and stored at – 80 °C until analysis for the evaluation of antioxidant enzymes. Nine spleen samples were taken per group and immersed in the RNAlater solution (Sigma-Aldrich, Poole, UK) and stored at − 80 ℃ for gene expression. For residue analyses, brain, gills, liver, kidney, and muscles were collected and stored at − 80 ℃.

### Biochemical analysis in serum

The serum clinico-biomarkers of liver and kidney functions and lipid profiles were assayed using the commercial diagnostic kits. Transaminases (AST and ALT), and alkaline phosphatase (ALP) activities were assayed by methods of Reitman and Frankel (1957) and Roy (1970), respectively**.** The methods of Bradford (1976) and Doumas et al. (1971) were used to determine the total protein (TP) and albumin (Alb) levels. Additionally, calculation of globulin (Glb) level, ALT/AST ratio, and albumin/globulin ratio (A/G ratio) was carried out. The method of Fawcett and Scott (1960) for analysis of the urea level and the kinetic method of Siest et al. (1985) with creatinine level were achieved. For the lipid profiles, the described methods by Allian et al. (1974), Bucolo and David (1973), and Friedwald (1972), respectively, were used to measure the total cholesterol (TC), triglyceride (TG), and high-density lipoprotein (HDL) cholesterol levels. Friedewald’s equation was used for calculating the concentration of low-density lipoprotein (LDL) and very-low-density lipoprotein (VLDL).

To determine how much departure from the mean was caused by pesticide use, calculations were made using equation of Mansour and Gamet-Payrastre (2016):

$$\%\;\mathrm{of}\;\mathrm{change}\;=\;(\mathrm{Treatment}\;\mathrm{value}-\mathrm{control}\;\mathrm{value})/\mathrm{control}\;\mathrm{value}\;\mathrm X\;100$$

Additionally, the amelioration index (AI), which represented the ameliorative impact of MOE, could be assessed by comparing a specific biochemical parameter's results in the ABM + MOE and control groups by using Mansour and Gamet-Payrastre (2016) formula:

$$\mathrm{Amelioration}\;\mathrm{index}(\mathrm{AI})=\lbrack\mathrm{Treatment}\;\mathrm{value}\;(\mathrm{ABM}+\mathrm{MOE})/\mathrm{control}\;\mathrm{value}\rbrack$$

### Evaluation of immunological parameters

The turbidity assay was used to evaluate lysozyme activity (LYSO) according to the method described by Ellis (1990). Spectrophotometric measurement of nitrous oxide (NO) was conducted using Griess reagent (Sigma-Aldrich, USA) (Anderson 1964). According to the manufacturer’s protocol, an immunoglobulin M (IgM) commercial ELISA kit (catalog number CSB-E12045Fh; CUSABIO, China) was used to measure IgM (mg per dl) at 450 nm.

### Oxidative stress and antioxidant enzyme assays

Liver and brain tissues were homogenized in ice-cold 50 mM sodium phosphate buffer (pH: 7) containing 0.1 mM ethylenediaminetetraacetic acid (EDTA) yielding 10% (W/V) homogenate. The homogenates were centrifuged at 12.000 g for 30 min at 4 °C, and the supernatants were aliquoted and kept at − 40 °C for oxidative stress and antioxidant enzyme assays. The concentration of thiobarbituric acid reactive products (malondialdehyde, MDA) was used to estimate lipid peroxidation (Ohkawa et al. 1979). Superoxide dismutase (SOD), and glutathione-s-transferees (GST) activity was measured by the methods of Marklund and Marklund (1974) and Habig et al. (1974), respectively. Total glutathione (GSH) content and glutathione peroxidase activity (GPx) were examined by Beutler et al. (1963). According to Mansour and Gamet-Payrastre (2016), alteration in the levels of biochemical parameters due to ABM was determined. On the other hand, the “amelioration index” (AI) was estimated as previously mentioned in biochemical parameters evaluation.

### Analysis of TGF-β, TNF-α, IL-1β, and IL-10β mRNA gene expression

The spleen samples were taken and cleaned in PBS buffer (pH 7.2). The easy-RED kit (iNtRON Biotechnology, South Korea) was used to extract total RNA from spleen tissue according to the instructions. The QuantiTect Reverse Transcription kit (Qiagen, Germany) was used in accordance with the manufacturer’s instructions to produce cDNA from a total of 1 µg of RNA. Table 2 lists the primer sequences used to amplify the β-actin, transforming growth factor-β (TGF-β), tumor necrosis factor (TNF-α), interleukin 1 beta (IL-1β), and interleukin 10 beta (IL-10β) genes (Qiang et al. 2014; Standen et al. 2016) and cycling condition using an Applied Biosystems™ 7500 Real-Time PCR (Thermo Fisher Scientific, USA). According to the standard curve, the amplification effectiveness of each primer was more than 97%. After normalizing the qRT-PCR results against the β -actin reference gene, relative expression was assessed using the ^2−ΔΔ^CT method (Yuan et al. 2006). The results of each experimental group were compared to the control and were expressed as fold changes.

**Table 2:** Primers sequences, target genes, and cycling conditions for RT-PCR

Target gene	Primers sequences	Product size (bp)	Reverse transcription	Primary denaturation	Amplification (40 cycles)			Dissociation curve (1 cycle)			Reference
					Secondary denaturation	Annealing (Optics on)	Extension	Secondary denaturation	Annealing	Final denaturation	
β-actin (EU887951)	CCACACAGTGCCCATCTACGA CCACGCTCTGTCAGGATCTTCA	89	50 °C 30 min	94 °C 5 min	94 °C 15 s	62 °C 30 s	72 °C 30 s	94 °C 1 min	62 °C 1 min	94 °C 1 min	Qiang et al. 2014
TGF-β (XM_003459454)	GTTTGAACTTCGGCGGTACTG TCCTGCTCATAGTCCCAGAGA	80	50 °C 30 min	94 °C 5 min	94 °C 15 s	60 °C 30 s	72 °C 30 s	94 °C 1 min	60 °C 1 min	94 °C 1 min	Standen et al. 2016
IL-1β (XM_005457887)	TGGTGACTCTCCTGGTCTGA GCACAACTTTATCGGCTTCCA	86	50 °C 30 min	94 °C 5 min	94 °C 15 s	62 °C 30 s	72 °C 30 s	94 °C 1 min	62 °C 1 min	94 °C 1 min	Standen et al. 2016
IL-10β (XM_003441366)	CTGCTAGATCAGTCCGTCGAA GCAGAACCGTGTCCAGGTAA	94	50 °C 30 min	94 °C 5 min	94 °C 15 s	60 °C 30 s	72 °C 30 s	94 °C 1 min	60 °C 1 min	94 °C 1 min	Standen et al. 2016
TNF-α (AY428948)	CCAGAAGCACTAAAGGCGAAGA CCTTGGCTTTGCTGCTGATC	82	50 °C 30 min	94 °C 5 min	94 °C 15 s	60 °C 30 s	72 °C 30 s	94 °C 1 min	60 °C 1 min	94 °C 1 min	Standen et al. 2016

### Residue analysis in tissues

Abamectin residues were extracted by adding acetonitrile (5:1 v/w) to well-homogenized fish tissues (gills, brain, liver, and kidney muscles), and clean-up was carried out by 25 mg C18 and 150 mg anhydrous magnesium sulfate according to Anastassiades et al. (2003). A UV detector set at 260 nm was used to examine ABM residues using HPLC (high-performance liquid chromatography) (Agilent 1260). Column Eclipse XDB-C 18 (5 µm, 4.6*250 mm) and methanol/water (9:1, v/v) as the mobile phase at a flow rate of 1 ml/min were employed. These conditions resulted in good separations, and high sensitivity was obtained. To assay the efficacy of the used extraction, clean-up, and a final determination procedure, recovery estimate was carried by untreated fish tissues as described by Mounes et al. (2020).

### Statistical analysis

The obtained data are represented as mean ± standard error (M ± SE). The significance of the difference between the tested groups was calculated by one-way ANOVA followed by Duncan’s test at *P* ≤ 0.05 using the IBM Statistical Package for Social Science for Windows (WINSPSS) version 25 (Chicago, USA). Shapiro–Wilk W test and homogeneity of variances were used to check the data for normality.

## Results

### Characterization of MOE

The total phenol content (TPC) of the ethanolic extract obtained from *Moringa oleifera* leaf powder was 106 ± 3 mg GAE g^−1^ dry extract, while the total flavonoid (TFCs) content was 58 ± 1.6 mg QE g^−1^ dry extract (Table 3). The major phenolic and flavonoid compounds in MOE were determined by HPLC. Cinnamic acid has the highest concentration (11 mg/g dry extract) of all the components.

**Table 3:** Major phenolic and flavonoid compounds in *Moringa oleifera* leaf ethanolic extract estimated by HPLC

Compounds	Retention time (min)	Concentration (mg/g dry extract)
Gallic acid	7	2
Chlorogenic acid	6.5	1.7
Catechin	16	0.9
Caffeic acid	6.64	5
Cinnamic acid	10.5	11
Syringic acid	19.8	5.5
Rutin	8.2	6
Naringenin	15	10
Quercetin	10.3	6
*Total phenol contents (TPCs)*	106 ± 3 mg GAE g^−1^ dry extract	
*Total flavonoid contents (TFCs)*	58 ± 1.6 mg QE g^−1^ dry extract	

### Clinical symptoms and post-mortem findings

**Fig. 2:**
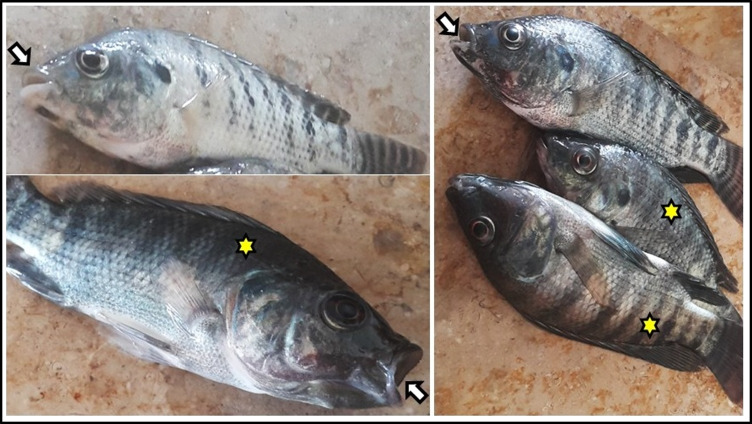
Main symptom of fish from ABM group (fish fed with control reference diet and exposed to 0.5 ug/l of abamectin (ABM 5% EC) showing open mouth (white arrow) and darkness of coloration (yellow star)

The CONT and MOE groups exhibited no behavioral changes or clinical signs, and both groups had a 100% survival rate. Contrarily, the ABM-exposed group had the highest mortality rate (30%) during the experiment with the emergence of clinical symptoms, the most significant of which were surface swimming, loss of appetite, and darkening of the skin color. Fish lost some of their reflexes, such as becoming less responsive to knocking on the aquarium wall and more susceptible to net capture, especially during the trial’s last week. Most of the fish had been dead with their mouths open (Table 4 and Fig. 2). However, the results indicated that the addition of MOE in the diet of ABM + MOE group ameliorated the effects of ABM via diminishing of mortality rate (6.67%) and severity of clinical symptoms, as shown in Table 4.

**Table 4:** Effect of phenolic-rich *Moringa oleifera* leaf extract on survival and clinical symptoms of *Oreochromis niloticus* exposed to abamectin (5% EC) for 28 days

Clinical signs	Experimental groups				
		CONT	ABM	MOE	ABM + MOE
No. of fish that survived	*No = 30	30 (100%)	21 (70%)	30 (100%)	28 (93.33%)
Rapid and surface swimming	No	0/30 (0%)	9/21 (42.85%)	0/30 (0%)	2/28 (7.14%)
	Score	−	+ +	−	+
Low of appetite and food intake	No	0/30 (0%)	15/21 (71.42%)	0/30 (0%)	2/28 (71.42%)
	Score	−	+ + +	-	+
Loss of reflexes (knocking on one side of the aquaria and escape when try to catch fish)	No	0/30 (0%)	10/21 (47.61%)	0/30 (0%)	3/28 (10.71%)
	Score	−	+ +	−	+
Respiratory manifestation (rapid operculum movement and opening mouth to gasp air)	No	0/30 (0%)	10/21 (47.61%)	0/30 (0%)	1/28 (3.57%)
	Score	−	+ +	−	+

^*^ No = number of fish/experimental group

The symptoms were observed, and their score were established as follow: ( −) no, ( +) weak, (+ +) moderate, and (+ + +) severe

*CONT*, control group where fish were fed with control reference diet without any exposure; *ABM*, fish fed with control reference diet and exposed to 0.5 ug/l of abamectin (ABM 5% EC, Profery); *MOE*, fish fed with phenolic-rich *Moringa oleifera* leaf extract-supplemented diet without any exposure; *ABM* + *MOE*, fish fed with phenolic-rich *Moringa oleifera* leaf extract-supplemented diet and exposed to 0.5 ug/l of abamectin (ABM 5% EC, Profery)

### Biochemical parameters

The biochemical parameters are displayed in Table 5, which illustrates a significant increase in serum ALT, AST, total protein (TP), and albumin (Alb) levels while there is a significant decrease in serum ALP activities in the ABM group in comparison to control. However, in ABM + MOE group, ALT, AST, ALT/AST ratio, and ALP levels were decreased, while TP, Alb, and A/G ratio levels were increased. The serum ALT, AST, and ALP activities were shown to be improved by MOE, with ameliorative index (AI) readings of 0.82, 0.94, and 0.92, respectively. In addition, fish in MOE group showed an increase in ALT, ALT/AST, and A/G when compared to the control group.

**Table 5:** Effect of phenolic-rich *Moringa oleifera* leaf extract on liver functions of *Oreochromis niloticus* exposed to abamectin (5% EC) for 28 days

Parameters	CONT	ABM	MOE	ABM + MOE	%Change	AI
ALT (U/l)	10.59 ± 0.22^c^	15.69 ± 0.50^a^	11.74 ± 0.11^b^	8.73 ± 0.20^d^	48.12	0.82
AST (U/l)	45.35 ± 1.83^b^	72.62 ± 1.26^a^	46.08 ± 0.62^b^	42.95 ± 0.24^c^	60.12	0.94
ALT/AST	0.23 ± 0.01^b^	0.22 ± 0.01^bc^	0.25 ± 0.01^a^	0.20 ± 0.004^c^	− 11.98	0.92
ALP (U/l)	16.53 ± 0.39^a^	14.55 ± 0.39^b^	15.59 ± 0.36^ab^	15.26 ± 0.48^ab^	––	––
TP (g/dl)	4.08 ± 0.04^b^	4.72 ± 0.03^a^	3.94 ± 0.06^b^	4.59 ± 0.07^a^	––	––
Alb (g/dl)	1.04 ± 0.01^c^	1.53 ± 0.02^a^	1.07 ± 0.01^c^	1.25 ± 0.01^b^	––	––
Glb (g/dl)	3.04 ± 0.04^bc^	3.19 ± 0.05^ab^	2.87 ± 0.07^c^	3.33 ± 0.06^a^	––	––
A/G	0.34 ± 0.01^c^	0.48 ± 0.01^a^	0.37 ± 0.01^b^	0.37 ± 0.01^b^	––	––

Means followed by different letters within each row are significantly different according to Duncan test (*P* ≤ 0.05). Data are presented as mean ± SE

Abbreviations: *ALT*, alanine transaminase; *AST*, aspartate transaminase; *ALP*, alkaline phosphatase; *TP*, total protein; *Alb*, albumin; *Glb*, globulin, *AI*, ameliorative index

CONT = control group where fish were fed with control reference diet without any exposure; *ABM*, fish fed with control reference diet and exposed to 0.5 ug/l of abamectin (ABM 5% EC, Profery); *MOE*, fish fed with phenolic-rich *Moringa oleifera* leaf extract-supplemented diet without any exposure; *ABM* + *MOE*, fish fed with phenolic-rich *Moringa oleifera* leaf extract-supplemented diet and exposed to 0.5 ug/l of abamectin (ABM 5% EC, Profery)

Regarding the lipid profile, all treatments (ABM and ABM + MOE) significantly increased the fish’s serum levels of total cholesterol, triglycerides, LDL, and VLDL, while there was reduction in HDL levels when compared to the control group (*P* ≤ 0.001). Also, supplementation with MO extract induced rise in level of serum total cholesterol when compared with the control group (Table 6).

**Table 6:** Effect of phenolic-rich *Moringa oleifera* leaf extract on lipid profile of *Oreochromis niloticus* exposed to abamectin (5% EC) for 28 days

Parameters	CONT	ABM	MOE	ABM + MOE
T. Cholesterol (mg/dl)	67.70 ± 0.70^d^	143.40 ± 2.96^a^	73.38 ± 0.25^c^	91.10 ± 1.38^b^
Triglyceride (mg/dl)	84.77 ± 1.68^c^	119.90 ± 1.53^a^	85.86 ± 1.30^c^	109.70 ± 2.33^b^
HDL (mg/dl)	35.29 ± 1.09^a^	27.16 ± 0.50^b^	36.37 ± 0.57^a^	26.44 ± 0.68^b^
LDL (mg/dl)	15.45 ± 0.81^c^	92.27 ± 2.36^a^	19.84 ± 0.61^c^	42.71 ± 1.83^b^
VLDL (mg/dl)	16.95 ± 0.33^c^	23.97 ± 0.30^a^	17.17 ± 0.26^c^	21.95 ± 0.46^b^

Means followed by different letters within each row are significantly different according to Duncan test (*P* ≤ 0.05). Data are presented as mean ± SE

Abbreviations: *T. Cholesterol*, total cholesterol; *HDL*, high-density lipoprotein; *LDL*, low-density lipoprotein; *VLDL*, very-low-density lipoprotein; *CONT*, control group where fish were fed with control reference diet without any exposure; *ABM*, fish fed with control reference diet and exposed to 0.5 ug/l of abamectin (ABM 5% EC, Profery); *MOE*, fish fed with phenolic-rich *Moringa oleifera* leaf extract-supplemented diet without any exposure; *ABM* + *MOE*, fish fed with phenolic-rich *Moringa oleifera* leaf extract-supplemented diet and exposed to 0.5 ug/l of abamectin (ABM 5% EC, Profery)

**Fig. 3:**
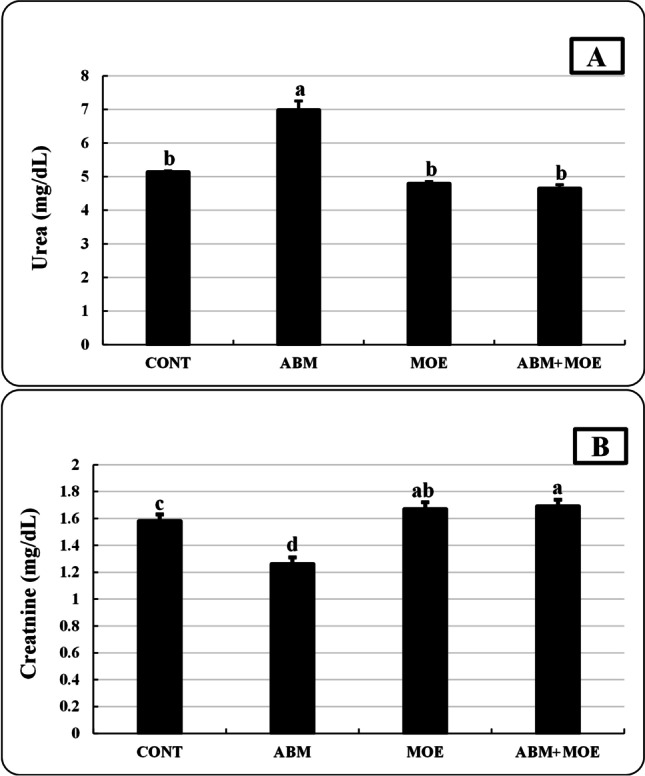
Effect of phenolic-rich *Moringa oleifera* leaves extract on urea (**A**) and creatinine (**B**) of *Oreochromis niloticus* exposed to abamectin (5% EC, Profery) for 28 days. Data are presented as mean ± SE. The bars with different letters are significantly different according to Duncan test (*P* ≤ 0.05). CONT, control group, where fish were fed with control reference diet without any exposure; ABM, fish fed with control reference diet and exposed to 0.5 ug/l of abamectin (ABM 5% EC, Profery); MOE, Fish fed with phenolic-rich *Moringa oleifera* leaf extract supplemented-diet without any exposure; ABM + MOE, fish fed with phenolic-rich *Moringa oleifera* leaf extract-supplemented diet and exposed to 0.5 ug/l of abamectin (ABM 5% EC, Profery)

On the other hand, the results demonstrated in Fig. 3 indicate that fish exposed to ABM had higher urea levels but lower creatinine concentration, while the opposite result was recorded in ABM + MOE.

### Immunological parameters

**Fig. 4:**
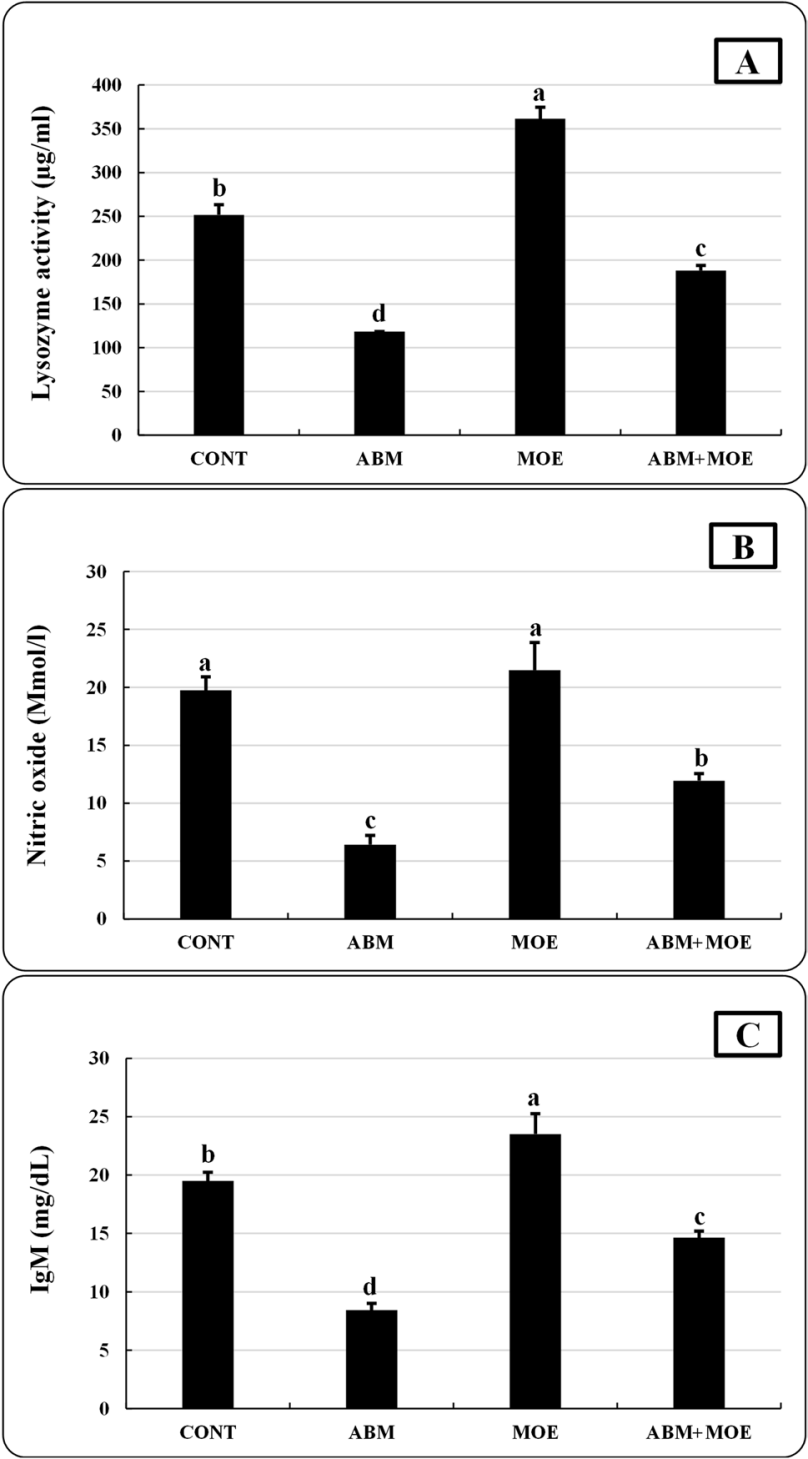
Effect of phenolic-rich *Moringa oleifera* leaf extract on lysozyme activity (**A**), nitric oxide (**B**), and IgM (**C**) of *Oreochromis niloticus* exposed to abamectin (5% EC, Profery) for 28 days. Data are presented as mean ± SE. The bars with different letters are significantly different according to Duncan test (*P* ≤ 0.05). CONT, control group, where fish were fed with control reference diet without any exposure; ABM, fish fed with control reference diet and exposed to 0.5 ug/l of abamectin (ABM 5% EC, Profery); MOE, fish fed with phenolic-rich *Moringa oleifera* leaf extract-supplemented diet without any exposure; ABM + MOE, fish fed with phenolic-rich *Moringa oleifera* leaf extract-supplemented diet and exposed to 0.5 ug/l of abamectin (ABM 5% EC, Profery)

Nitric oxide activity was not significantly different in the MOE group compared to the control group, whereas IgM and lysozyme activity was significantly higher in the MOE group. On the other hand, there was a significant reduction in lysozyme activity, nitric oxide, and IgM in the ABM group compared to the control. Treatment with MOE reduced the negative effects of ABM on immunological parameters in the ABM + MOE group of fish, but the measured parameters did not reach the levels seen in the control group (Fig. 4).

### Antioxidant capacity

In comparison to the control group, liver samples from the ABM group had significantly higher levels of MDA in both the brain and the liver, as well as SOD, GPx, GST, and GSH, while the GPx in brain samples and the GSH in liver and brain samples considerably decreased in the group subjected to ABM (Table 7). There was no discernible variation in SOD and GST levels across the brain samples from the various experimental groups. On the other hand, MOE dietary supplementation mitigated the oxidative stress induced by ABM exposure, where MDA and GSH levels in brain and liver samples and SOD, GPx, and GST levels in liver samples were significantly improved.

**Table 7:** Effect of phenolic-rich *Moringa oleifera* leaf extract on brain and liver oxidative stress and antioxidants of *Oreochromis niloticus* exposed to abamectin (5% EC) for 28 days

Parameters	organ	CONT	ABM	MOE	ABM + MOE	% Change	AI
MDA (nmol/g tissue)	Brain	11 ± 0.98^b^	28.77 ± 1.99^a^	11.06 ± 0.53^b^	10.61 ± 0.45^b^	161.6	0.96
	Liver	10.62 ± 0.32^b^	17.40 ± 0.80^a^	9.36 ± 0.70^b^	11.20 ± 0.73^b^	63.87	1.05
SOD (U/min/g tissue)	Brain	172.20 ± 0.59	170.40 ± 0.93	169 ± 0.41	171.50 ± 1.27	− 1.033	0.99
	Liver	168.70 ± 4.60^b^	186.50 ± 4.4^a^	167 ± 2.72^b^	165.80 ± 3.1^b^	10.59	0.983
GPx (nmol/g tissue)	Brain	3136 ± 101.80^a^	2842 ± 98.4^b^	3280 ± 35.79^a^	2675 ± 101.20^b^	− 9.348	0.853
	Liver	2385 ± 197.70^b^	2957 ± 175.50^a^	2485 ± 160.40^b^	2445 ± 36.8^b^	23.98	1.025
GST (µmol/min/g tissue)	Brain	1460 ± 219.80	1547 ± 224.50	1419 ± 126.60	1649 ± 97.88	5.896	1.12
	Liver	4539 ± 218.70^c^	7051 ± 373.50^a^	4580 ± 264.30^c^	5674 ± 283.40^b^	55.33	1.250
GSH (nmol/g tissue)	Brain	3448 ± 151.10^a^	1756 ± 96.2^b^	3400 ± 148.20^a^	3363 ± 175.40^a^	− 49.08	0.97
	Liver	2642 ± 206.10^a^	1885 ± 130.70^b^	2774 ± 162.90^a^	3043 ± 110.07^a^	− 28.66	1.151

Means followed by different letters within each row are significantly different according to Duncan test (*P* ≤ 0.05). Data are presented as mean ± SE

Abbreviations: *MDA*, malondialdehyde; *SOD*, superoxide dismutase; *CAT*, catalase; *GPx*, glutathione peroxidase; *GST*, glutathione-s–transferees; *GSH*, total glutathione; *AI*, ameliorative index; *CONT*, control group where fish were fed with control reference diet without any exposure; *ABM*, fish fed with control reference diet and exposed to 0.5 ug/l of abamectin (ABM 5% EC, Profery); *MOE*, fish fed with phenolic-rich *Moringa oleifera* leaf extract-supplemented diet without any exposure; *ABM* + *MOE*, fish fed with phenolic-rich *Moringa oleifera* leaf extract-supplemented diet and exposed to 0.5 ug/l of abamectin (ABM 5% EC, Profery)

### Relative expression of immune-related genes

**Fig. 5:**
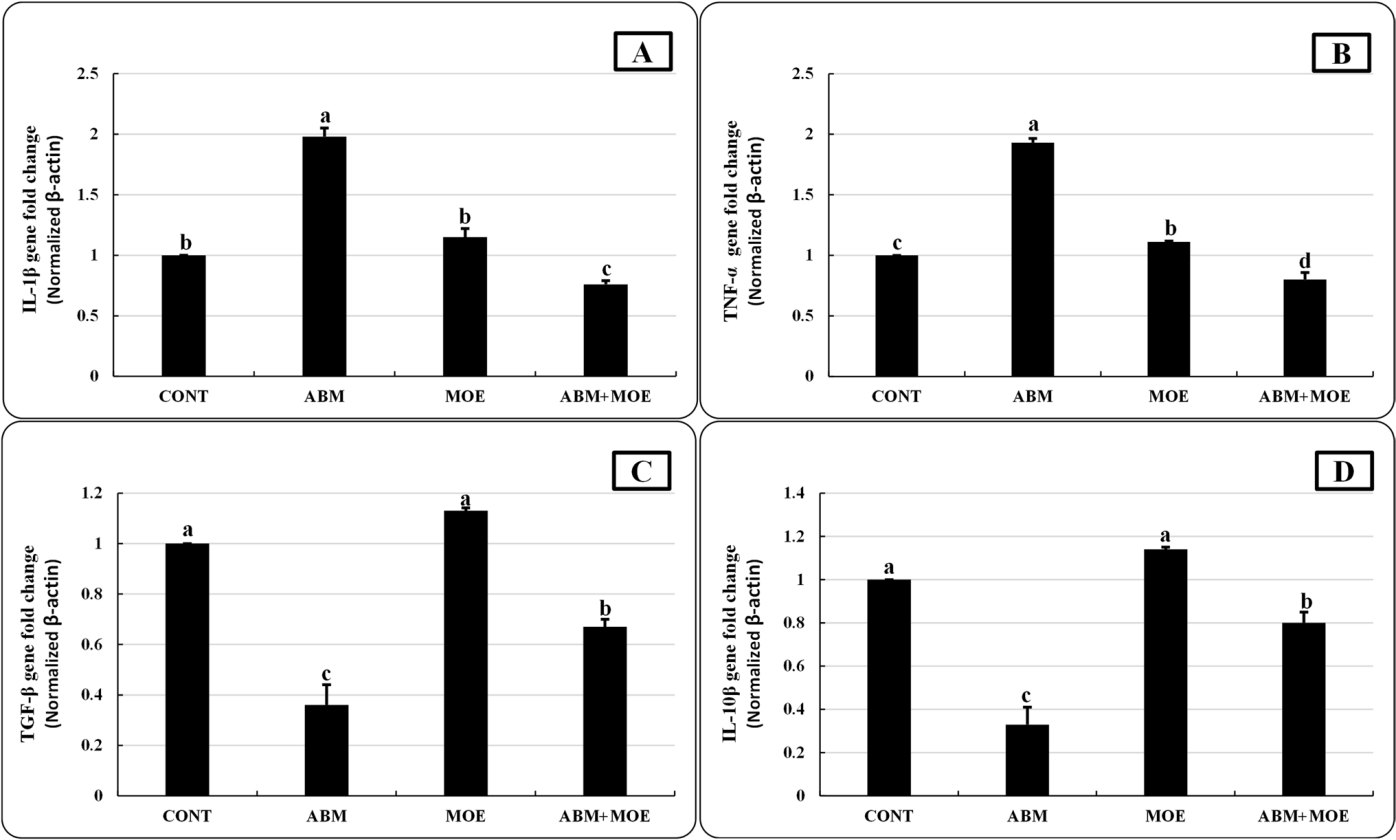
Effect of phenolic-rich *Moringa oleifera* leaf extract on interleukin-1β (IL-1β) (**A**), tumor necrosis factor α (TNFα) (**B**), transforming growth factor beta (TGF-β) (**C**), and interleukin-10β (IL-10β) (**D**) of *Oreochromis niloticus* exposed to abamectin (5% EC, Profery) for 28 days. Data are presented as mean ± SE. The bars with different letters are significantly different according to Duncan test (*P* ≤ 0.05). CONT, control group, where fish were fed with control reference diet without any exposure; ABM, fish fed with control reference diet and exposed to 0.5 ug/l of abamectin (ABM 5% EC, Profery); MOE, fish fed with phenolic-rich *Moringa oleifera* leaf extract-supplemented diet without any exposure; ABM + MOE, fish fed with phenolic-rich *Moringa oleifera* leaf extract-supplemented diet and exposed to 0.5 ug/l of abamectin (ABM 5% EC, Profery)

As shown in Fig. 5, an estimation of the relative levels of expression of the immune-related genes was obtained using RT-PCR analysis of the mRNA levels of the genes. There were no significant differences in the level of IL-1β, TGF-β, and IL-10β mRNA between the MOE and control groups, while the mRNA of TNF-α level was significantly upregulated (1.11-fold) in the MOE group in comparison to CONT. On the other hand, in the ABM-exposed group, the levels of IL-1β and TNF-α mRNA were markedly up-regulated (1.98- and 1.93-fold, respectively), while TGF-β and IL-10β mRNA levels were down-regulated (0.36- and 0.33-fold, respectively). In the ABM + MOE group, the TGF-β and IL-10 anti-inflammatory genes were up-regulated (0.67- and 0.8- fold, respectively), while the IL-1β and TNF-α inflammatory genes were down-regulated (0.76- and 0.8-fold, respectively).

### Residue levels of fish tissue

Three spiking levels with five replicates for each level resulted in recovery percentages that ranged from 77 to 93.8%. By creating a calibration curve using mean standards from five injections, linearity was assessed. It was determined that the correlation coefficient (*R*^2^) was 0.999.

The results in Table 8 show that ABM residues in muscles, gills, brain, liver, and kidney fish samples were 1.70, 3.00, 5.3, 3.2, and 2.8 ppb, respectively, whereas the amount of ABM residues fed on diet supplement with MOE reduced. The highest detectable levels of ABM residues were detected in brain tissues, but the lowest were in muscles. The relative standard deviation (% RSD), which is the relationship between standard deviation and average concentration discovered, has been identified as the measure of precision. The RSD (%) ranged from 1.9 to 5.5 with the tested pesticide and 2.4 to 6.3 in fish exposed to ABM + MOE. The low detection limit (LOD) and limit of quantitation (LOQ) of ABM were determined to be 0.01 and 0.06 mg/kg, respectively.

**Table 8:** Residue levels of abamectin (5% EC) alone and with *Moringa oleifera* leaf extract in different fish (*Oreochromis niloticus)* tissues after 28 days of exposure

Organs	ABM (5% EC)		ABM + MOE	
	Residues (ppb)	RSD (%)	Residues (ppb)	RSD (%)
Muscle	1.70 ± 0.11	2.7	1.50 ± 0.20	2.4
Gills	3.00 ± 0.10	1.9	1.60 ± 0.10	2.7
Brain	5.30 ± 0.30	4.9	1.30 ± 0.20	2.6
Liver	3.20 ± 0.20	5.5	2.10 ± 0.30	6.3
Kidney	2.80 ± 0.40	2.5	1.80 ± 0.30	3.3

Data are presented as mean ± SD

Abbreviations: *ppb*, parts per billion; *RSD (%)*, the relative standard deviation; *ABM*, fish fed with control reference diet and exposed to 0.5 ug/l of abamectin (ABM 5% EC, Profery); *ABM* + *MOE*, fish fed with phenolic-rich *Moringa oleifera* leaf extract-supplemented diet and exposed to 0.5 ug/l of abamectin (ABM 5% EC, Profery)

## Discussion

According to Environmental Protection Agency Office of Pesticide Programs (EPA/OPP) and the Globally Harmonized System of Classification and Labelling of Chemicals (GHS), ABM is classified as very high toxic compound (category 1), which is extremely toxic to aquatic species. For example, the 96 h-LC_50_ of abamectin (Profery 5% EC) for *Oreochromis niloticus* is 10 ug/l (Farag and Reda 2021).

Herbal and medicinal plants have recently received a lot of attention because they contain various chemicals (e.g., phenolic and flavonoid compounds) that have antibacterial, anticancer, anti-inflammatory, antioxidant, and immunostimulant properties (Tungmunnithum et al. 2018). The results of the current study confirmed that MOE contains high levels of total phenols and flavonoids, which was supported by the results of Sreelatha and Padma (2009), Moyo et al. (2012), and El-Seadawy et al. (2022).

In the current results, the ABM-exposed group had the highest rate of mortality (30%), along with a variety of clinical symptoms including loss of appetite, skin darkening, and loss of some reflexes, similar to our results, Dos Santos et al. (2023) showed a reduced escape capacity, lack of feeding, and browning in zebra fish exposed to ABM even at acute or chronic exposure. After 38 and 24 h of exposure to ABM at 3.0 and 4.5 μg/l, *Oncorhynchus mykiss* stayed to the bottom of aquariums with sluggish movement and dark pigmentation in their coloring (Jenčič et al. 2006). The skin darkening and behavioral changes may be related to the neuroendocrine stress response to ABM, which affects brain monoaminergic activity and could be responsible for all the physiological and behavioral changes in fish (Øverli et al. 2001). In the present study, MOE displayed a potential impact on the attenuation to the ABM toxicity symptoms in *O. niloticus.* This reduction in toxicity symptoms will be explained by all of the analyses in this study as well as the changes generated by the MOE supplementation, particularly its antioxidant and immune-boosting qualities.

Liver is a detoxification organ that serves as a biomarker for environmental pollution (Norhan et al. 2022). ALT is a sensitive indication of hepatocyte impairment and might be raised even when no symptoms are present. AST can be detected in the liver, skeletal muscle, kidney, brain, and erythrocytes. ALT activity is lower in extrahepatic tissues, making it a more specific indication of liver damage than AST (Kulkarni et al. 2021). Therefore, ALT, AST, and ALP testing can be utilized for aquatic pollution biomonitoring (de la Torre et al. 2000; Vaglio and Landriscina 1999). Even slight cellular damage can be detected by an increase in these enzymes’ activity in the extracellular fluid or serum (Coeurdacier et al. 2011; Patriche et al. 2011). On the other hand, the total serum globulin level is considered an indicator of general specific immunity in fish (Yılmaz and Ergün 2018).

According to our findings, fish intoxicated by ABM significantly increased their serum levels of ALT, AST, TP, and Alb. These results are explained by Hsu et al. (2001) and Castanha Zanoli et al. (2012) who reported that ABM intoxication disrupts hepatocyte function and causes liver damage in addition to impairing neuronal coordination. In addition, the interaction of the ABM with the adenine nucleotide translocator may contribute to the toxicity of the hepatocytes by functionally inhibiting the translocator and impairing mitochondrial bioenergetics (Castanha Zanoli et al. 2012). Similar to our results, Rohmah et al. (2022) reported that *Cyprinus carpio* exposed to 12.5% LC_50_ of ABM for 30 days had a substantial increase in serum ALT. Similarly, plasma ALT and AST increased in 96-h ABM-exposed *O. niloticus* (Fırat and Tutus 2020). On contrary, AST and ALT were significantly declining in white snail, *Theba pisan*, after 7 days of exposure to 20 and 60% LD_50_ ABM doses (48 h-LD_50_ value of 1.048 μg/snail) (Radwan and Gad 2022). Kushwaha et al. (2020) reported a substantial rise in the AST/ALT ratio in the *O. mossambicus* group subjected to 55 ppb of ABM for 48 h, and they attributed this finding to the fibrotic injury to liver tissue caused by ABM exposure.

ALP activity was not statistically substantially increased in *O. mossambicus* exposed to 40 and 45 ppb of ABM for 48 h, but it was considerably increased in the high-dose group (55 ppb for 48 h) due to the biliary blockage caused by ABM exposure (Kushwaha et al. 2020). The decreased activity of alkaline phosphatase in pesticide-exposed fish may be connected to the interactions between pesticides and co-factors and regulators, as well as its inducement to cell organelle damage such as the endoplasmic reticulum and membrane transport system damage (Al-Ghanim et al. 2020). On the contrary, the *Labeo rohita* juveniles fed with emamectin benzoate ≥ 250 μg kg^−1^ of fish biomass per day for 21 days had considerably high levels of ALP (Choudhary et al. 2022). The marked increase in serum total protein, albumin, and globulin was recorded in *O. niloticus* exposed to ABM acute toxicity at 5 µg/l for 96 h (Farag and Reda 2021). In another study, abamectin was found to dramatically lower total protein concentration in tilapia fish tissues, including the liver, muscle, kidney, and gills (Al-Kahtani 2011). *O. niloticus* subjected to 1/10 of the ABM 96-h LC_50_ (20.73 µg/l) for 84 days demonstrated a considerable drop in globulin levels because of the ABM-induced liver damage and dysfunction (Mahmoud et al. 2021). Notably, several other studies supported our findings (Abou-Zeid et al. 2018; Mobeen et al. 2022; Thiripurasundar et al. 2014), indicating that ABM has the potential to damage the liver.

In this study, the MOE supplementation diminished the hepatotoxic effect of ABM in the ABM + MOE group by enhancing ALT, AST, TP, Alb, and Glb levels and A/G ratio. This hepatoprotective effect of MOE could be attributed to the presence of quercetin (Table 3) as a protective agent for liver diseases, as quercetin inhibited liver inflammation primarily via NF-B/TLR/NLRP3, mTOR activation in autophagy, and inhibited the expression of apoptotic factors associated with the development of liver diseases as recorded by Zhao et al. (2021). Furthermore, the MOE contains caffeine and cinnamic acids, which when coupled with triphenyl phosphonium cations, protect the hepatic mitochondria from lipid peroxidation, apoptosis, and a decline in hydrogen peroxide levels (Espíndola et al. 2019; Li et al. 2017). The previously reported hepatoprotective properties of MOE components could have resulted in liver cell membrane stability and stop the leakage of enzymes (Pari and Karthikesan 2007; Toppo et al. 2015). Toppo et al. (2015) demonstrated that oral supplementation with MO extract (500 mg/kg daily for 28 days) mitigated the hepatotoxic effect of cadmium in rats. Numerous studies have shown that MO has hepatoprotective properties against a variety of contaminants, including ABM (Abdelrasoul 2018), pendimethalin (Hamed and El-Sayed 2019), chlorpyrifos (Ibrahim et al. 2019), carbon tetrachloride (Selvakumar and Natarajan 2008), 7,12- dimethyl-benz[a]anthracene (Sharma and Paliwal 2012), and cadmium (Toppo et al. 2015).

In the current study, Nile tilapia in the ABM group had significantly higher levels of total cholesterol, triglycerides, LDL, and VLDL during the experimental exposure period. Similar to our results, Mahmoud et al. (2021) reported that the lipid profile of *O. niloticus* subjected to ABM (20.73 μg/l) for 84 days was significantly affected except HDL value. Moreover, El-Said (2007) indicated that hypercholesterolemia was detected in abamectin-exposed *O. niloticus* (at 50.48 and 103. 68 μg/l) after 14 days of exposure. The elevation of the ABM group lipid profile could be attributed to the damage of hepatic cell membranes, which allowed the leakage of total cholesterol, triglycerides, LDL, and VLDL (El-Shenawy 2010). Hypercholesterolemia could be explained by an enhanced production by the liver (and other organs) or release of cholesterol-damaged cell membranes, and/or this may be due to inhibit the conversion of cholesterol into steroid sex hormones (Mukherjee et al. 1991; Singh and Reddy 1990).

The current study has proven the palliative effect of MOE against the toxic effect of ABM on the *O. niloticus* lipid profile in ABM + MOE group. This palliative effect of MOE could be related to chlorogenic acid, which has anti-dyslipidemic properties and characteristics by lowering plasma total cholesterol and triglycerides (Cho et al. 2010). However, phenolic compounds, saponins, and flavonoids in MOE play significant roles in lipid regulation by inhibiting pancreatic cholesterol esterase activity, delaying and decreasing cholesterol absorption, and binding bile acids by forming insoluble complexes and increasing their fecal excretion, which lowers plasma cholesterol levels (Adisakwattana and Chanathong 2011; Oyedepo et al. 2013).

The two main biochemical markers used to evaluate renal health are creatinine and urea (Gounden et al. 2022). Urea and creatinine are the byproduct of protein catabolism, so the elevation in urea concentration may be caused by ABM impact on liver or kidney function (Nasr et al. 2016). In the present study, the fish exposed to 0.5 ug/l ABM exhibited a significant increase in the urea levels and reduction of the creatinine concentration. This is in line with the findings of Farag and Reda (2021) who found that fish exposed to ABM (5% EC) had considerably higher serum urea and lower creatinine levels after 96 h. Since the kidney is the organ that largely excretes urea, the increase in urea concentration may be linked to decreased kidney glomerular filtration, dysfunctional kidney tubules, and impaired kidney function (Magdy et al. 2016; Walmsley and White 1994). On contrary, Mahmoud et al. (2021) recorded a significant increase in *O. niloticus* exposed to 20.73 μg/l ABM for 84 days. Also, on day 14, the creatinine level in ABM-exposed *O. niloticus* (103.68 µg/l) increased (El-Said 2007). Our results and those of various experiments may differ due to differences in fish life stages, ABM concentrations, and time of exposure. From these results, we can conclude that ABM could boost biochemical catabolism to meet the increased energy demand of stressed animals or could decrease synthesis due to impaired tissue function (Magdy et al. 2016).

On the other hand, MOE supplementation minimized the disruptive effect of ABM on renal function in the ABM + MOE co-treated group, demonstrating a move toward normal. When MO leaves were added to the diet of *O. niloticus* that had been exposed to dimethoate, the levels of urea and creatinine were brought back to normal compared to the control, especially after 30 days (Soror et al. 2021). The MO leaf extracts could protect kidneys from the effects of toxicity by strengthening the body’s natural antioxidant system to withstand the environment of oxidative stress through various naturally bioactive components and highly accessible essential trace elements (vitamin A, nicotinic acid, vitamin B, citric acid, malic acid, succinic acid, fumaric acid, and oxalic acid) in this extract (Karthivashan et al. 2016; Sharma and Paliwal 2012).

The immune health condition of aquatic animals is negatively impacted by the watery pesticide and any other environmental contaminants, and has an impact on these aquatic species’ survival and growth rates as well as their resistance to infections (Cuesta et al. 2011). In the current study, ABM caused an immunosuppressive effect on *O. niloticus* by decreasing lysozyme and nitric oxide activates and IgM in comparison to the control group. These results are similar to those of Hong et al. (2020b) for *Eriocheir sinensis*, Mahmoud et al. (2021) for *O. niloticus*, and Rohmah et al. (2022) for *Cyprinus carpio* despite the fact that each of them used a different exposure dose and duration. The present study revealed that dietary supplements of MOE could improve the hazardous effect of ABM on LYSO, NO, and IgM of ABM + MOE group. This enhancement may be due to the presence of MOE cinnamic acid and other phenolic and flavonoid components as well as naringenin that act as immunomodulators (Havsteen 2002; Kaurinovic and Vastag 2019; Salehi et al. 2019). The findings are consistent with earlier research by Ibrahim et al. (2019) and Mahmoud et al. (2022), which indicated that MOE protected *O. niloticus* from the immunosuppressive effects of chlorpyrifos and fipronil.

Even though aquatic organisms can produce free radicals and reactive oxygen species (ROS) naturally during their aerobic metabolism, they may increase in stressful environments due to exposure to hazardous substances such as ABM (Ajima et al. 2021). The oxidative damage resulting from the increase in ROS could affect aquatic organisms’ physiological functions and impair their immunological status. The current findings reveal that 28 days of exposure to 0.5 ug/l ABM increased levels of MDA in the brain and liver, as well as SOD, GPx, and GST in liver samples, which explains the causes of disturbance in liver and kidney functions in the ABM group in this study. The results are in line with previous research that showed that ABM can induce oxidative stress and alter the antioxidant response in several fish and shellfish species such as *O. niloticus* (Mahmoud et al. 2021; Mansour et al. 2022), *Cyprinus carpio* (Rohmah et al. 2022), *Schizothorax prenanti* (Hong et al. 2020a), and *Eriocheir sinensis* (Hong et al. 2020b). Furthermore, the current research showed that dietary supplementation with MOE mitigated the adverse effects of ABM on the antioxidant system of exposed fish in ABM + MOE group. The relative abundance of polyphenols (phenyl propanoids, phenolic acids, flavonoids, and tannins) in *M. oleifera*, which function as hydrogen donors that stabilize free radicals produced by cells via electron donation or acceptance, directly contributes to MOE antioxidant effect in order to improve *O. niloticus* antioxidant response (Sreelatha and Padma 2009).

Cytokines are tiny proteins that influence the formation of all blood cells as well as other cells that aid in the immunological and inflammatory responses of the body. In the present results, ABM elevated the inflammatory genes (TNF-α and IL-1β) and declined the anti-inflammatory genes (TGF-β and IL-10β). These findings may be related to cell damage and inflammatory reactions created by ABM, as well as inflammation caused by increased ROS production, which increases inflammatory cytokines by stimulating NF-_k_B pathway activation (Gur et al. 2022). The findings are consistent with a prior report on rats given ABM orally at a dose of 1 mg/kg every other day for 28 days, which markedly elevated the expression levels of NF-_k_B, IL-1β, and TNF-α in testicular tissue (Gur et al. 2022). In the current investigation, the mRNA levels of inflammatory genes in the spleen tissues of ABM + MOE fish were significantly lower than in the ABM group alone. The downregulation of inflammatory cytokines by MOE supplementation in the ABM + MOE group could be attributed to an increase in anti-inflammatory gene expression. Additionally, MOE was found to block the upstream of mitogen-activated protein kinase activation, resulting in the downregulation of inflammatory mediator expression (Muangnoi et al. 2012).

Predicting potential harmful effects of the compounds is easier with the knowledge of their distribution in organisms and liposolubility. It is generally known that some compounds build up in compartments within the environment and within living things (Paraíba et al. 2009). Fish, therefore, absorb insecticides after coming into contact with them in water. The majority of pesticides readily pass through biological membranes due to their lipophilicity, which makes fish more susceptible to aqueous insecticides. Insecticides may then get bioconcentrated in various fish tissues. Given that fish are lower on the food cycle, bioaccumulation of pesticides may rise in the tissues of their predators and consumers such as humans, having an adverse effect on the health and survival of these individuals (Lushchak et al. 2018; Rani et al. 2022). Therefore, the bioaccumulation of these pollutants in fish and the possible biomagnifications in humans are considered to be a hazard to hepatotoxic and neurologic injuries as well as reproductive disorders (Paraíba et al. 2009).

In the current study, the highest detectable levels of ABM residues were detected in brain and then liver tissues, but the lowest were in muscles. Various enzymatic alterations result from hazardous substance accumulation in the liver that exceeds the tolerable level (Al-Ghanim et al. 2020). The organism’s ability to absorb and disperse toxicants depends on both the toxicant and the organism. Examples of these factors include the compound’s hydrosolubility, liposolubility, and degree of ionization, octanol–water partition coefficient, and tissue lipid contents among others (Paraíba et al. 2009). ABM has been discovered to collect at elevated levels in fatty tissue, such as the brain, liver, and fat, because of its strong lipophilic nature (González Canga et al. 2009). In the present study, MOE efficiently removed ABM residues from fish tissues. MOE had the greater potential effect to remove ABM from brain samples (from 5.3 to 1.3 ppb), while it has a small potential effect to remove ABM from muscles (from 1.7 to 1.5 ppb). The acceptable relative standard deviation in our result was between 1.9 and 6.3%, which is in agreement with that published by European Commission Directorate General for Health and Food Safety (ECDGHFS 2015). Therefore, *M. oleifera* ethanolic extract (MO) can efficiently remove abamectin residues from fish. MO is an environmentally friendly natural coagulant and able to treat water containing undesirable toxicant materials (Soumaoro et al. 2021), so the decreased ABM concentration in exposed fish can be explained by the process of coagulation with coagulants present in MO. Moreover, MOE could reduce ABM accumulation in different fish tissues due to a potential chelating activity by some substances in MOE and/or through facilitating the ABM excretion from the body (Nakata et al. 2022). Further investigations are required to clarify the mechanism by which MO reduced the pesticide and heavy metal accumulation in the different tissues.

## Conclusion

The current investigation supports the promising role of *Moringa oleifera* as a potential natural detoxifying agent in counteracting the hepato-renal toxic effects of abamectin exposure and limiting its accumulation in fish musculature and other tissues. However, more research is required to investigate the precise mechanisms by which *Moringa oleifera* produces its ameliorative actions in fish.

## Data Availability

All data generated or analyzed during this study are included in this published article.
